# General practitioners' attitudes and preparedness towards Clinical Decision Support in e-Prescribing (CDS-eP) adoption in the West of Ireland: a cross sectional study

**DOI:** 10.1186/1472-6947-10-2

**Published:** 2010-01-12

**Authors:** Chee Peng Hor, James M O'Donnell, Andrew W Murphy, Timothy O'Brien, Thomas JB Kropmans

**Affiliations:** 1 Department of Medicine, National University of Ireland, Galway, Clinical Science Institute, Galway, Ireland; 2 Department of Pharmacology (retired), National University of Ireland, Galway, Clinical Science Institute, Galway, Ireland; 3 Department of General Practice, National University of Ireland, Galway, Clinical Science Institute, Galway, Ireland

## Abstract

**Background:**

Electronic clinical decision support (CDS) is increasingly establishing its role in evidence-based clinical practice. Considerable evidence supports its enhancement of efficiency in e-Prescribing, but some controversy remains. This study evaluated the practicality and identified the perceived benefits of, and barriers to, its future adoption in the West of Ireland.

**Methods:**

This cross sectional study was carried out by means of a 27-part questionnaire sent to 262 registered general practitioners in Counties Galway, Mayo and Roscommon. The survey domains encompassed general information of individual's practice, current use of CDS and the practitioner's attitudes towards adoption of CDS-eP. Descriptive and inferential analyses were performed to analyse the data collected.

**Results:**

The overall response rate was 37%. Nearly 92% of respondents employed electronic medical records in their practice. The majority acknowledged the value of electronic CDS in improving prescribing quality (71%) and reducing prescribing errors (84%). Despite a high degree of unfamiliarity (73%), the practitioners were open to the use of CDS-eP (94%) and willing to invest greater resources for its implementation (62%). Lack of a strategic implementation plan (78%) is the main perceived barrier to the incorporation of CDS-eP into clinical practice, followed by i) lack of financial incentives (70%), ii) lack of standardized product software (61%), iii) high sensitivity of drug-drug interaction or medication allergy markers (46%), iv) concern about overriding physicians' prescribing decisions(44%) and v) lack of convincing evidence on the systems' effectiveness (22%).

**Conclusions:**

Despite favourable attitudes towards the adoption of CDS-eP, multiple perceived barriers impede its incorporation into clinical practice. These merit further exploration, taking into consideration the structure of the Irish primary health care system, before CDS-eP can be recommended for routine clinical use in the West of Ireland.

## Background

The introduction of electronic prescribing (e-Prescribing) a decade ago, whether adopted alone or functionally incorporated into electronic medical record (EMR) regimens, has transformed prescribing practice. The proposed incorporation of clinical decision support (CDS) mechanisms such as formulary prescription, drug-drug interaction checking and drug allergy checking, into e-Prescribing provides a means of optimizing the prescribing process, including stewardship for prescribing decisions and enhancement of the safety and appropriateness of a prescription [1]. It potentially improves the communication pathway between prescribers and dispensers, as well as augmenting the cost-effectiveness of national healthcare planning [1,2].

There is considerable evidence supporting the role of CDS within e-Prescribing (CDS-eP) in enhancing prescribing efficiency [1,3-6], however some controversy over its design, operational functions and national implementation remain [1,7-9].

In the Irish primary care setting, despite increasing application of electronic medical records (EMR), the functions of e-Prescribing have only been partially adopted and utilized. Currently, the majority of general practitioners (GPs) utilize the paper mechanisms such as the British National Formulary (BNF), Monthly Index of Medical Specialties Ireland (MIMS) and international or local guidelines, as a reference to support their prescribing decision. Meanwhile, some practitioners employ electronic mechanisms such as prescribing websites and software products as an additional reference. To date, an acceptable and user-friendly CDS-eP has yet to be developed for the Irish primary care system. However, recent announcements from the Irish Health Service Executive (HSE) indicated an extension in its Information Technology (IT) strategy to include IT-related projects, a national electronic prescription system and EMR implementation [10,11]; this reflects a continued progress in the Irish e-Health initiative and may provide opportunity for further research in developing an optimal CDS-eP for primary care.

Prior to implementing a new initiative such as CDS-eP, thorough assessment of the involved parties is essential. As prescribers are key players, their understanding, attitudes and acceptance of this initiative play a decisive role in determining the success of its implementation. The current developing trend has therefore led us to initiate this study with specific aims:

1. to assess, among GPs in the West of Ireland,

a. the prevalence of EMR adoption

b. the current use of CDS mechanism(s) in their prescribing practice

2. to identify,

a. their perceived benefits of CDS-eP adoption in future

b. the potential barriers impeding its implementation

c. their presumptive responses towards potential alerts flagged by CDS-eP

## Methods

### Survey instrument

This study was carried out using a cross sectional survey among GPs in the West of Ireland. A 27-question survey was developed and evaluated through a pilot study with five GPs to assess its comprehension and appropriateness. These questions were formulated with reference to the selected publications [8,9,12,13], reviewed for their relevance and subsequently categorized into two sections in the survey.

A short explanatory note was incorporated into the survey to provide a standardized reference for the respondents in understanding the two main concepts central to this study. They were(1) "e-Prescribing (eP) allows prescribers to utilize electronic systems to facilitate and enhance the communication of a prescription, aiding the choice, administration or supply of a medicine through decision support and providing a robust audit trail for the entire medicine use process," and (2) "Clinical Decision Support within the e-Prescribing context (CDS-eP) is an algorithm that establishes the safety and appropriateness of a prescription, with links to a third party information system employed to enhance the safety of the prescription. Such systems may include clinical checks which allow alerts to be flagged up to the prescribers on drug-drug interactions or formulary status" [7].

Section I evaluated the individual GP's practice, including their practice duration in general practice, practice type and practice premise, prevalence of EMR adoption and current use of CDS mechanism(s) in their prescribing practice.

Section II comprised three components to assess the GPs for (i) the benefits they perceived towards CDS-eP adoption in their clinical practice, (ii) the potential barriers they perceived impeding its implementation in the primary care setting and (iii) their presumptive responses to potential alerts flagged by CDS-eP. The respondents were asked to rate their responses for each statement using ordinal five-level Likert items [14]. This survey did not specifically refer to any software currently in use for e-Prescribing or CDS-eP.

The survey was ended with an open question for any free comments on CDs-eP from the respondents.

### Study participants

The questionnaire together with a one-page of personalized introductory cover letter was sent to 262 registered public and private GPs in Counties Galway, Roscommon and Mayo, representing a complete sample group for this survey. The postal details were obtained from the HSE database, which captured all GPs in the counties. However, the data source did not include any information that would provide any description of the GPs who would respond to this survey. The respondents were given an option whether to remain anonymous or to identify themselves in this survey. A pre-paid return envelope was provided to enhance responses [15]. This study took place between 10^th ^July and 31^st ^August 2008. The study was granted the ethical approval from the University Research Ethics Committee of the National University of Ireland, Galway.

### Data analysis

All the collected data were compiled into a database and further analysed using Statistical Package for the Social Sciences (SPSS) version. 14.0. In addition to the descriptive analyses, different inferential statistical analyses were performed. The ordinal five-level Likert items (strongly agree, agree, neither, disagree and strongly disagree) were collapsed to three data points (agree, neither, disagree) for inferential statistical analyses. Using Pearson Chi square (χ^2^) tests, we examined the associations (a) between prevalence of EMR adoption and practice premise, (b) between current use of CDS mechanisms and perceived value of different CDS mechanisms in assisting GPs' prescribing decision, and (c) between the GPs' preparedness towards future CDS-eP adoption, and duration in general practice (data were categorized into two categories according to median general practice), practice premise and current use of CDS mechanisms. If the assumptions for a χ^2 ^test were not met, Fisher's exact or linear by linear association test would be applied as an alternative test. Besides, Spearman's correlation tests were performed to examine the relationship between the different aspects of GPs' preparedness, and their perceived barriers towards future CDS-eP adoption. In addition, we investigated whether the duration of individual GP's practice influenced the frequency of EMR adoption, their preparedness and their perceived barriers towards CDS-eP adoption using one-way Anova tests. The level of statistical significance for all inferential analyses was defined at p-value less than 0.05.

An additional component to survey "the GPs' feelings when the alerts flagged by CDS-eP during the prescribing process" was reported incomprehensive and not answered by majority of the respondents. It was hence omitted from data analysis.

## Results

We obtained an overall response rate of 37% (98 out of 262) in this study. Nearly 45% (44 out of 98) of respondents identified themselves in this survey.

### Overview of individual practice and EMR prevalence

The practice durations of the respondents were variable, ranging from one to 39 years. Half of them had at least 19 years of experience in the field of general practice. There were nearly equal proportions of GPs currently working in either city (30.9%) or rural (32%) areas, while 37.1% had a mixed practice. Approximately 60% of them practiced in a group of at least two whole-time equivalent GPs, with the remaining 40% operating single-handedly. Among those practising in groups, half practiced in a group of two whole-time equivalent practitioners, followed by 31% in a group of three, 13% in a group of four, 3% in a group of five and 2% in a group of eight.

**Figure 1:**
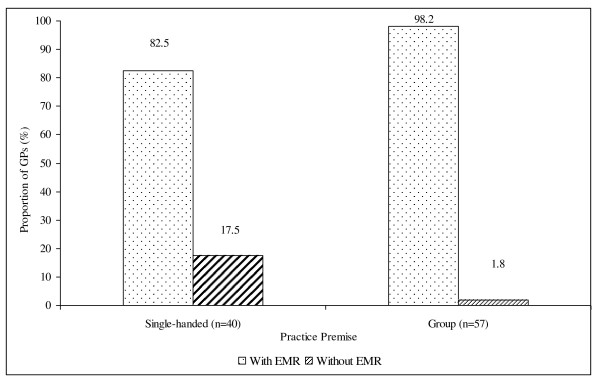
**Adoption of EMR among GPs in single-handed and group practices**.

Ninety-two percent of the respondents had adopted an EMR system in their practice, chosen from these software products available in Ireland - HEALTH *one*™, Medicom^® ^or GP Mac. There was a high prevalence of EMR adoption among both single-handed and group-practice GPs. However, there is a significant correlation between EMR adoption and practice premises, whereby approximately 18% of single-handed GPs still employed the conventional paper system in keeping the consultation record for their patients, as compared to 2% of group-practice GPs (Fisher exact test, p = 0.008) (Figure 1). However, their duration of practice did not seem to influence the frequency of EMR adoption in their practice.

### Current use of CDS mechanisms and their values in prescribing process

With regard to the types of CDS mechanisms presently used by the GPs in their prescribing practice, 45% of them relied solely on the conventional paper mechanisms, such as BNF, MIMS, and local or international guidelines. Only 5% of GPs referred to computer or internet-based information exclusively. The remaining half of the respondents utilized combinational electronic and paper mechanisms in supporting their prescribing decisions. In addition, two respondents reported referring to the specialists in hospital for prescribing advice occasionally.

**Figure 2:**
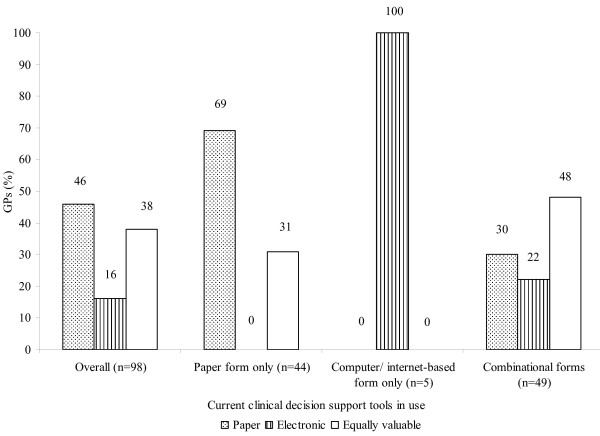
**GPs' evaluation on different CDS mechanisms in assisting their prescribing decision**.

Forty-six percent of the GPs considered paper mechanisms to be superior to electronic CDS mechanisms in assisting their prescribing practice. Almost 38% recognized electronic mechanisms as equally valuable to the paper mechanisms as a decision support tool in their prescribing practice. There was a significant correlation between the current CDS mechanisms in use by the GPs and their evaluation of these mechanisms (Linear by Linear Association, p = 0.017) (Figure 2). Although the majority who solely employed paper mechanisms valued more the paper mechanisms, one third perceived that electronic mechanisms were not inferior to the paper mechanism. Among respondents who currently utilized the combinational mechanisms, as many as 70% acknowledged the value of electronic CDS mechanisms in guiding their prescribing decision.

### Attitudes and preparedness towards CDS-eP adoption

Seventy percent of the respondents believed that CDS-eP has the capacity to improve quality, while nearly 84% considered that using this new mechanism may reduce prescribing errors (Table 1). About one in five respondents were concerned that this mechanism may reduce their decision making power in prescribing. Ninety-four percent of GPs expressed their readiness to learn and use this new mechanism, and more than 60% were willing to invest greater resources in its adoption in future. Seventy-three percent of the respondents were unfamiliar with what CDS-eP is and how it is used in clinical prescribing practice. There was no statistically significant correlation between the GPs' preparedness towards future CDS-eP adoption, and their practice duration, practice premises or current use of CDS mechanisms.

**Table 1:** GPs' attitudes and preparedness towards CDS-eP

Statements	Agree	Neutral	Disagree
I am familiar with what CDS-eP is and how it is used in clinical prctice (N = 93)	27%	12%	61%
I believe that CDS-eP has the capacity to improve prescribing quality (N = 91)	71%	28%	1%
I believe that using CDS-eP may reduce prescribing errors (N = 91	84%	14%	2%
I believe that CDS-eP may reduce my decision making power in prescribing (N = 90)	19%	32%	49%
I am open to learning/using new CDS-eP (N = 95)	94%	4%	2%
My practice is willing to invest greater resources in CDS-eP in the future (N = 92)	62%	26%	12%

N -- number of respondents, excluding missing responses. Total respondents = 98

### Potential barriers impeding CDS-eP adoption

Among the six major pre-selected barriers, the top three barriers ranked by at least 60% of respondents were beyond individual capacity of control. The lack of a strategic national implementation plan was perceived as the main obstacle for future implementation of CDS-eP in their clinical practice (Table 2), followed by the lack of financial incentives and of acceptable, standardized software products. Among the subsequent three barriers specific to the prescribers, high sensitivity of drug-drug interaction and medication allergy markers present within CDS-eP was the main concern. Forty-four percent expressed concern regarding their degree of flexibility to override the suggestions or decision made by CDS-eP. One in five respondents considered the lack of convincing evidence on the mechanism's effectiveness as an impediment towards its future adoption. There was no statistically significant correlation between the perceived barriers and the GPs' preparedness towards the adoption of such new mechanism.

**Table 2:** Potential barriers impeding implementation of CDS-eP in general practice

Perceived barriers	Agree	Neutral	Disagree
Lack of convincing evidence regarding its effectiveness (N = 88)	22%	44%	34%
High sensitivity of drug -- drug interaction or drug allergy markers (N = 85)	46%	41%	13%
Concern about the degree of flexibility for the physician to override CDS-eP (N = 88)	44%	32%	24%
Lack of financial incentives (N = 87)	70%	24%	6%
Lack of acceptable, standardized product software (N = 88)	61%	33%	7%
Lack of a strategic plan for implementation (N = 89)	78%	20%	2%

N -- number of respondents, excluding missing responses. Total respondents = 98

### Presumptive responses towards alerts from CDS-eP

**Figure 3:**
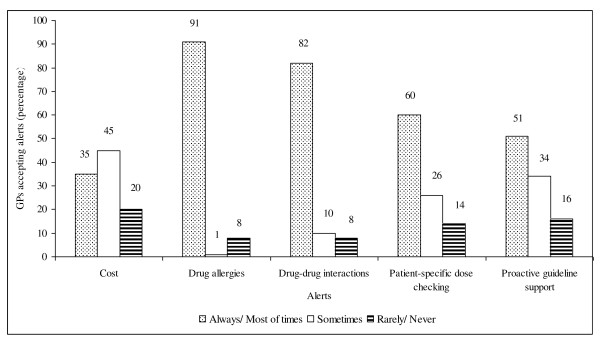
**Presumptive responses towards potential alerts flagged by CDS-eP during prescribing process**.

Medication allergies (91%) and drug-drug interaction (82%) flagged by CDS-eP would be the two alerts accepted by the majority of GPs most of the times during the prescribing process, while a cost-related alert would be the least prioritized element (Figure 3).

### General comments on CDS-eP

A total of 13 comments were provided by the respondents with some illustrated in the Additional File 1. Concern was expressed regarding the potential interference with their prescribing independence, as well as the system's efficiency as an up-to-date clinical decision support tool. With regards to hypersensitivity of drug-drug interaction markers, a GP commented that "really depend(s) on the specificity e.g. I know that angiotensin-converting enzyme (ACE) inhibitors and diuretics interact. This may be why I prescribe them (e.g. for enhanced antihypertensive effect or to avoid ACEI-induced hyperkalaemia). I probably would get frustrated if the alert is flagged every time I attempt to co-prescribe these medication and especially if the alert substantially delayed my prescribing (e.g. by 20 seconds)".

## Discussion

In this study, majority of the GP respondents displayed positive attitudes towards the potential benefits brought by CDS-eP. They expressed their readiness to embrace the new mechanism through their willingness to learn and to invest greater resources in adopting this mechanism. In addition, the high prevalence of EMR adoption in the clinical practice has set a potential platform for future incorporation of CDS-eP into e-Prescribing system in order to optimize its functions. Moreover, there is an increased utilization of electronic CDS mechanisms in the Irish primary care setting. The positive value of such mechanisms in aiding their prescribing decision-making process has been acknowledged by the GPs. An increased confidence in the application, together with improved availability and accessibility to electronic sources may have contributed to emergence of this transitional phase, from relying solely on paper mechanisms to start exploring the electronic mechanisms. The assessment of the source of the specific electronic mechanism was beyond the scope of this study, and remains as an interesting aspect to explore further. Also, the high rates of appreciation for the value of CDS mechanisms and willingness for further investment may be specific to the sub-group of GPs who were likely to respond to this survey.

Worldwide, inappropriate prescribing in the community setting, particularly among elderly populations has been reported, with some resulting in adverse medication events [16-18]. Currently, there is no national programme or policy in place to collect and evaluate data related to medication errors in the Irish primary care setting. A report regarding the medication safety scheme from Tallaght Hospital in Dublin revealed that nearly 15% of 102 prescribing errors in the community setting reported involved patient harm [19]. Besides, prescribing errors accounted for 10 - 20% of legal claims against GPs in the United Kingdom and Ireland [20,21].

Since its inception, incorporation of CDS-eP into e-Prescribing systems has encountered challenges ranging from national standardized implementation to individual prescriber endorsement for its application in the clinical setting. The absence of a strategic national action plan was perceived as the greatest barrier of all. Further progress is awaited with the recent HSE announcements on its plan to invest in a national e-Prescribing system and IT-related projects [10,11]. Financial incentives often act as a driving force to accelerate adoption of the new initiative. Its long term implication for cost-saving would be significant. An example is a recent initiative by the Department of Health and Human Services in United States to launch an incentive payments scheme to eligible professional successful electronic prescribers, via Medicare over a five-year period from 2009. It is expected to save up to US $156 m by avoiding adverse medication events over the course of the programme [2]. McMullin et. al. reported that application of e-Prescribing systems with an integrated CDS mechanism in primary care had shifted the prescribing behaviour away from the high cost therapies and lowered prescription costs. The savings from the altered prescribing behaviours offset the subscription cost of the system [22]. More studies are needed to illustrate the cost-and-benefit analysis of CDS-eP adoption to convince authorities that investment is worthwhile in the primary care setting. Designation of standardized, acceptable and user-friendly software products for CDS-eP is a demanding process, especially with increasing emphasis on evidence-based prescribing [23].

Hypersensitivity of the drug allergy or drug-drug interaction alerts and concern over flexibility as a prescriber to override CDS-eP are the GP-specific barriers. Hypersensitivity of alerts is often attributed to frequent flagging of trivial or unnecessary alerts and in high volume. For example, running an allergy or drug-drug interaction check against medication history instead of current medication regimen [24]. Studies have indicated 40 - 96% of electronic medication safety alerts were overridden by physicians [24-26]. Factors contributing to overriding the alerts were poor specificity and their low significance in clinical context, alerts overload interrupting workflows and constant time pressure in clinical practice. Moreover, known interactions with justifiable benefits greater than risks and patients' resistance to change have also led to non-adherence to the alerts [8,24-29]. Concern over CDS-eP interfering with the prescribers' decision making power, as expressed by one-fifth of GP respondents may become an inherent factor contributing to the resistance to adopting the mechanism in individual practice. It is proposed that allowing prescribers to set their desirable threshold of alerts severity may be expected to improve the alerts acceptance rate [24,28]. Also, a "smarter" system designed to stratify various medication alerts to their clinical relevance and utilize a set of mandatory alerts may improve prescribing safety [30]. In this study, more than 80% of GP respondents reported that they would accept drug allergy and drug-drug interaction alerts most of the time, if CDS-eP is in place.

There is considerable evidence supporting the roles of CDS-eP in enhancing prescribing quality [1,3-6]. The lack of training and exposure towards e-Prescribing-related issues may have contributed to the high degree of unfamiliarity among the GPs and their perception of lacking convincing evidence on the mechanism's effectiveness.

This study should be viewed with the following limitations is mind. A 37% response rate may have provided an unrepresentative sample that has limited views in this area. Besides, the survey was carried out among GPs in three out of 26 Irish counties, hence the results may not be generalizable to reflect the views of the entire Irish primary care. However this is the first study of its kind in Ireland and, at the very least, provides a valuable benchmark. Although there is a high EMR adoption rate (92%) among our respondents, future research should attempt to assess the attitudes and opinions of the non-respondents towards e-Health initiative.

We propose a multidisciplinary and multidimensional approach to make further progression with CDS-eP. At national level, establishment of an expert review panel specific to e-Prescribing and CDS-eP is needed to evaluate the current Irish e-Health initiatives (e.g. e-Prescribing system, e-transfer of medical information), assess in depth the existing barriers and draft practical recommendations for implementation. With a score of 42 points out of 100 for the e-Health component in the recent Euro Health Consumer Index (EHCI) 2008, more concerted efforts are needed by all stakeholders [31]. At local level, such as in the West of Ireland, a pilot feasibility study of CDS-eP adoption in selected practices is recommended.

## Conclusions

Prescribers' opinions and attitudes are a pre-requisite to determine the success of CDS-eP adoption in clinical practice. The results from this study reflect encouraging attitudes of GPs in the West of Ireland towards CDS-eP, potentially paving the way for its future adoption, as part of a better integrated care pathway for patients. Major barriers identified require to be overcome for future progression through multidisciplinary collaboration.

## Competing interests

The authors declare that they have no competing interests.

## Authors' contributions

CPH is an undergraduate medical student researcher who contributed to the questionnaire design, collected and analysed data and led the write-up of this paper. TJBK and JMOD are the research supervisors to CPH. Both of them, together with AWM and TOB had contributed to the study design and write-up of this study. All the authors have read and approved the final manuscript.

## Pre-publication history

The pre-publication history for this paper can be accessed here:

http://www.biomedcentral.com/1472-6947/10/2/prepub

## Supplementary Material

Additional file 1 **Additional comments from GP respondents on CDS-eP**. GPs' views with regards to their recognition and concerns on CDS-eP mechanism. Click here for file
