# Supplementary material for: General practitioners' attitudes and preparedness towards Clinical Decision Support in e-Prescribing (CDS-eP) adoption in the West of Ireland: a cross sectional study
Source: BMC Med Inform Decis Mak. 2010 Jan 12;10:2. doi: 10.1186/1472-6947-10-2 (PMC2824732; doi:10.1186/1472-6947-10-2)
Supplement: Additional file 1 — Additional comments from GP respondents on CDS-eP. GPs' views with regards to their recognition and concerns on CDS-eP mechanism. [file 1472-6947-10-2-S1.DOC]

| **Additional comments from GP respondents on CDS-eP** |
| --- |
| **Recognition towards CDS-eP** |
| “An important requisite for safe prescribing and quality assurance” |
|  |
| “Willing to learn and to get involved in CDS-eP…” |
|  |
| “Willing to invest depends on cost/ benefit analysis” |
|  |
|  |
| **Concerns towards CDS-eP design and efficiency** |
| “ a useful tool but not a full substitute for one’s own decision” |
|  |
| “…worried that my independence as prescriber(s) might be curtailed….” |
|  |
| “would want to feel confident that the information it throws up is reliable, validated, and updated regularly…” |
|  |
| “get frustrated if the alert substantially delayed my prescribing” |
|  |
| “ it has to be a smart system where the alert does not become intrusive, e.g. for a particular patient, I could ask the (system) to ignore a particular interaction alert” |
